# Prevalence of HBV and HCV Infections in Iranian Blood Donors; An Updated Systematic Review and Meta-Analysis

**DOI:** 10.34172/mejdd.2025.423

**Published:** 2025-04-30

**Authors:** Leila Kasraian, Mohammad Hossein Imanieh, Reza Tabrizi, Reza Shahriarirad, Amirhossein Erfani, Sahar Hosseini

**Affiliations:** ^1^Blood Transfusion Research Center, High Institute for Research and Education in Transfusion Medicine, Tehran, Iran; ^2^Iranian Blood Transfusion Organization, Shiraz, Iran; ^3^Gastroenterohepatology Research Center, Shiraz University of Medical Sciences, Shiraz, Iran; ^4^Non-Communicable Diseases Research Center, Fasa University of Medical Sciences, Fasa, Iran; ^5^Health Policy Research Center, Institute of Health, Shiraz University of Medical Sciences, Shiraz, Iran; ^6^Student Research Committee, Shiraz University of Medical Sciences, Shiraz, Iran

 This correction has been implemented in both the PDF and HTML versions of the article.

 This notice amends the article titled “Prevalence of HBV and HCV Infections in Iranian Blood Donors: An Updated Systematic Review and Meta-Analysis,” published in *Middle East Journal of Digestive Diseases* 2021; 13(3): 237-252 (DOI: 10.34172/mejdd.2021.231).

 The address of the first author was incorrectly listed in the main article and the author affiliation section. The correct address is:

 Blood Transfusion Research Center, High Institute for Research and Education in Transfusion Medicine, Tehran, Iran & Iranian Blood Transfusion Organization, Shiraz, Iran.

 This correction has been made to both the PDF and HTML versions of the article.

